# The role and utilisation of public health evaluations in Europe: a case study of national hand hygiene campaigns

**DOI:** 10.1186/1471-2458-14-131

**Published:** 2014-02-07

**Authors:** Jonathan R Latham, Anna-Pelagia Magiorakos, Dominique L Monnet, Sophie Alleaume, Olov Aspevall, Alexander Blacky, Michael Borg, Maria Ciurus, Ana Cristina Costa, Robert Cunney, Mojca Dolinšek, Uga Dumpis, Sabine Erne, Olafur Gudlaugsson, Dana Hedlova, Elisabeth Heisbourg, Jette Holt, Natalia Kerbo, Nina Kristine Sorknes, Outi Lyytikäinen, Helena C Maltezou, Stavroula Michael, Maria Luisa Moro, Christiane Reichardt, Maria Stefkovicova, Emese Szilágyi, Rolanda Valinteliene, Rossitza Vatcheva-Dobrevska, Natacha Viseur, Andreas Voss, Suzette Woodward, Laura Cordier, Andreas Jansen

**Affiliations:** 1 Office of the Chief Scientist, European Centre for Disease Prevention and Control, Tomtebodavägen 11A, SE-171 83 Stockholm, Sweden; 2 Ministère de la Santé, Paris, France; 3 Swedish Institute for Communicable Disease Control, Solna, Sweden; 4 Medical University of Vienna, Vienna, Austria; 5 Mater Dei Hospital, Valletta, Malta; 6 Medical University of Łódź, Łódź, Poland; 7 General Directorate of Health, Lisbon, Portugal; 8 Health Protection Surveillance Centre, Dublin, Ireland; 9 University Medical Centre Ljubljana, Ljubljana, Slovenia; 10 Stradins University Hospital, Riga, Latvia; 11 Office for Public Health, Vaduz, Liechtenstein; 12 Landspitali University Hospital, Reykjavik, Iceland; 13 Military Faculty Hospital Prague, Prague, Czech Republic; 14 Direction de la Santé, Luxembourg, Luxembourg; 15 Statens Serum Institut, Copenhagen, Denmark; 16 Health Board, Tallinn, Estonia; 17 Norwegian Institute of Public Health, Oslo, Norway; 18 National Institute for Health and Welfare, Helsinki, Finland; 19 Hellenic Center for Disease Control and Prevention, Athens, Greece; 20 Ministry of Health, Nicosia, Cyprus; 21 Institute for Hygiene and Environmental Medicine, Charité, Berlin, Germany; 22 Regional Public Health Authority in Trenčín, Trenčín, Slovakia; 23 Alexander Dubček University of Trenčín, Trenčín, Slovakia; 24 National Centre for Epidemiology, Budapest, Hungary; 25 Institute of Hygiene, Vilnius, Lithuania; 26 National Centre for Infectious and Parasitic Diseases, Sofia, Bulgaria; 27 Scientific Institute of Public Health, Brussels, Belgium; 28 Radboud University Nijmegen Medical Centre and Canisisus-Wilhelmina Hospital, Nijmegen, The Netherlands; 29 National Patient Safety Agency, London, UK; 30 London School of Hygiene and Tropical Medicine, London, UK

**Keywords:** Hand hygiene, Healthcare associated infections, Evaluation, Evidence-based public health

## Abstract

**Background:**

Evaluations are essential to judge the success of public health programmes. In Europe, the proportion of public health programmes that undergo evaluation remains unclear. The European Centre for Disease Prevention and Control sought to determine the frequency of evaluations amongst European national public health programmes by using national hand hygiene campaigns as an example of intervention.

**Methods:**

A cohort of all national hand hygiene campaigns initiated between 2000 and 2012 was utilised for the analysis. The aim was to collect information about evaluations of hand hygiene campaigns and their frequency. The survey was sent to nominated contact points for healthcare-associated infection surveillance in European Union and European Economic Area Member States.

**Results:**

Thirty-six hand hygiene campaigns in 20 countries were performed between 2000 and 2012. Of these, 50% had undergone an evaluation and 55% of those utilised the WHO hand hygiene intervention self-assessment tool. Evaluations utilised a variety of methodologies and indicators in assessing changes in hand hygiene behaviours pre and post intervention. Of the 50% of campaigns that were not evaluated, two thirds reported that both human and financial resource constraints posed significant barriers for the evaluation.

**Conclusion:**

The study identified an upward trend in the number of hand hygiene campaigns implemented in Europe. It is likely that the availability of the internationally-accepted evaluation methodology developed by the WHO contributed to the evaluation of more hand hygiene campaigns in Europe. Despite this rise, hand hygiene campaigns appear to be under-evaluated. The development of simple, programme-specific, standardised guidelines, evaluation indicators and other evidence-based public health materials could help promote evaluations across all areas of public health.

## Background

Public health programmes are increasingly being scrutinised and challenged to demonstrate efficacy and effectiveness, underscoring the growing importance and the need of evidence-based methodologies in public health [1]. One of the primary means of demonstrating success of a public health programme is through programme evaluation, which can be utilised to assess almost any aspect of a public health programme, from impact to cost-effectiveness. Evaluations are internationally acknowledged as an indispensable tool in guiding public health professionals and decision-makers in developing and augmenting public health programmes [2,3].

Hand hygiene is the single most effective way of reducing rates of healthcare-associated infections (HAIs) [4]. HAIs are defined by the World Health Organisation (WHO) as “infections acquired by patients while receiving care in healthcare settings” [5]. HAIs are of strategic importance because they result in high healthcare costs, increases in hospital length of stay and poorer patient outcomes [5]. The importance of hand hygiene is highlighted by data from European Centre for Disease Prevention and Control (ECDC), which estimate that HAIs affect about 7% of hospital admissions across the European Union [1]. In 2005 the WHO prioritised hand hygiene as an intervention of universal relevance across developed and developing countries with its First Global Patient Safety Challenge, Clean Care is Safer Care [6].

In 2009, ECDC demonstrated that hand hygiene campaigns were widely implemented throughout Europe, with 23 having implemented national or regional hand hygiene campaigns during the period 2000–2009 [7]. As part of the GPSC, the WHO developed consensus guidelines and a multimodal improvement and implementation strategy as well as a standardised self-assessment tool for healthcare facilities [8]. This tool uses standardised data collection methodologies and indicators for hand hygiene activities throughout the world. Although designed for use at the facility level, implementation at multiple sites can be used to evaluate effectiveness of regional or national campaigns. This publicly-available and ready-to-use rapid evaluation framework aims at facilitating the evaluation processes for hand hygiene campaigns. This is particularly important as hand hygiene interventions solicit behaviour change, thus addressing the complex interaction psychosocial, behavioural and religious factors which drive hand hygiene behaviours [9,10]. These psychosocial components have been found to affect outcomes in different environments [11-13], and the sustainability of achieved results [8,14] rendering evaluations for hand hygiene campaigns even more necessary.

The aim of this study was to identify the frequency and scope of evaluations of European national public health programmes, taking hand hygiene campaigns as an example. National hand hygiene campaigns performed in European Union (EU)/European Economic Area (EEA) Member States between 2000 and 2012 were considered.

## Methods

A cohort of national hand hygiene campaigns, which consisted of all national campaigns performed in the 29 EU/EEA Member States (as of January 2012) from January 2000 to December 2011 was utilised for the analysis. National campaigns that had taken place between January 2000- March 2009 in EU/EEA Member States were described in a previous publication from 2009 [7]. More recent campaigns (April 2009 to 31 December 2011) were identified by an online survey composed of structured and open-ended questions (see Additional file 1). The survey was distributed on 22 March 2012 via e-mail to nominated contact points for HAI surveillance in EU/EEA Member States. Respondents were given a two-week deadline to complete the survey and provide relevant documentation to ECDC.

Apart from identifying new campaigns, the 2012 survey was also used to collect information on both new and old campaigns initiated since 2000. Information sought included identifying the involvement of key stakeholders, sources of funding and specific activities undertaken. Furthermore the survey sought to collect data on any existing campaign evaluation, whether an evaluation had been performed, who was responsible, the source of funding for the evaluation, the involvement of stakeholders, the methodology employed and what indicators of success were utilised. If an evaluation had not been performed, the survey explored reasons for this, and whether an evaluation was planned for the future.

Data were entered into Microsoft Excel 2010. No statistical analysis was undertaken on the data. Text responses were grouped by keywords in order to identify and group major themes for open ended questions.

### Ethical Clearance

Ethical clearance was not required for the study as the data utilised is openly available.

## Results

The cohort used for analysis was composed of 36 campaigns in 20 EU/EEA Member States. Nineteen of the 36 campaigns were implemented in the 2000–2009 period and were identified in the 2009 study [7]. One of these campaigns was excluded from the analysis owing to an incomplete response on the questionnaire. The remaining 18 campaigns were identified during the current survey, and were implemented between April 2009 and 31 December 2011. The analysis was thus composed of 36 campaigns, 18 from 2000 – 2009 and 18 from April 2009 – December 2011. The results indicate an increase in the number of hand hygiene campaigns implemented annually in Europe over time.

**Figure 1:**
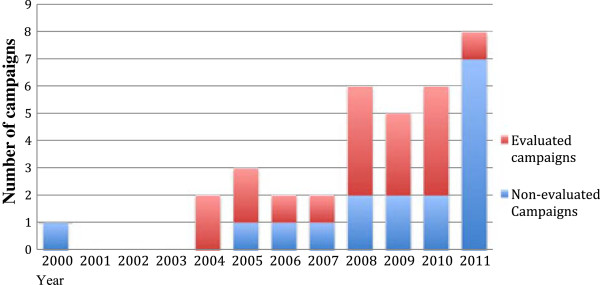
Number and evaluation status of hand hygiene campaigns by year implemented.

Ten countries reported having had no campaigns and a further ten countries had one campaign. Five countries had two campaigns, one had three, two countries had four and one had five. Of the 36 campaigns identified, 18 (50%) had undergone at least one evaluation (Figure 1). Sixty-seven percent of evaluations were run by the same organisation or ministry that had implemented the campaign. Public/Government money was used to fund all reported evaluations. Though this may have introduced a bias, we have no way to independently verify this. Ninety two percent of evaluations were developed with consultation from various stakeholders.

**Figure 2:**
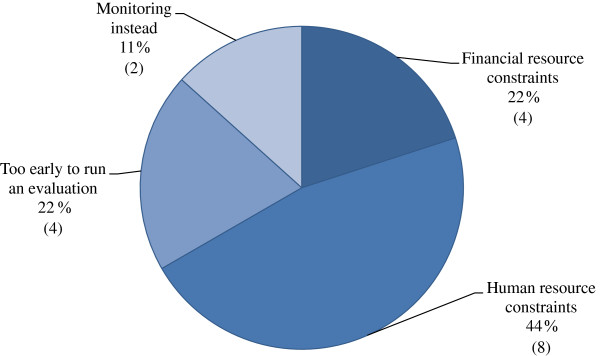
Reasons for non-evaluation of campaigns as percentages and total.

**Figure 3:**
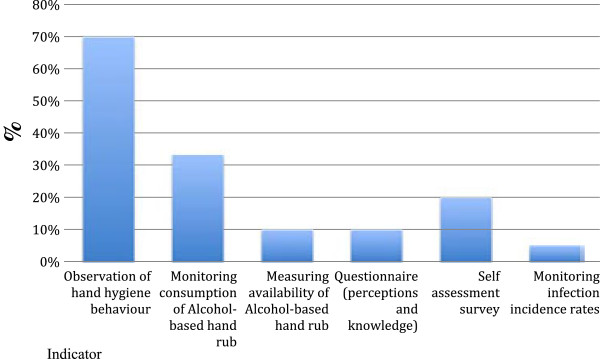
Methodologies and indicators utilised by evaluated campaigns (%).

Of the 50% of campaigns that had not been evaluated, 13% of respond indicated that permanent monitoring activities were underway and a further 20% that it was “too early in the campaign to initiate an evaluation”. The most commonly reported barrier to evaluating was ‘human resource constraints’ quoted by 47% of respondents (Figure 2). ‘Financial resource constraints’ were quoted by an additional 20% of respondents. Evaluation methodologies were grouped according to utilised evaluation indicators (Figure 3), with some evaluations employing multiple indicators. The most frequently used indicator was ‘direct observation of hand hygiene rates as a proportion of total opportunities’ followed by ‘monitoring total consumption of alcohol-based hand rub’ (Figure 3). Other indicators include assessing the ‘availability of alcohol-based hand rub’, ‘self-assessment surveys’, ‘a questionnaire’ and ‘monitoring of incidence rates of hygiene-associated notifiable diseases’. The WHO evaluation toolkit and self-assessment tool were widely utilised, with a reported 55% of all evaluated campaigns consulting or utilising the tool (this figure rises to 69% when excluding evaluations conducted before the publication of the tool) [8]. The remaining 45% of evaluations utilised a variety of indicators. Owing to the relative consensus on best practices for the assessment of hand hygiene activities however, evaluations, whether utilising WHO evaluation toolkits or not were similar.

More specifically, nine campaigns from four countries (Belgium, Greece, Italy and Portugal) reported specific changes in rates of hand hygiene compliance. The mean, increase in hand hygiene compliance for these campaigns was 15.4% per intervention. In countries where multiple interventions were implemented and evaluated, the first campaign was always associated with the highest rates of change. The largest single increase in hand hygiene compliance was reported following Greece’s 2010 campaign, which noted a 27.3% increase in hand hygiene compliance amongst nurses. Each subsequent intervention was associated with a lesser increase. Additionally, achieved compliance rates decreased rapidly post-intervention. This is consistent with findings from the literature [14].

By assessing the reported indicators, all evaluations identified positive outcomes and no ‘unsuccessful campaigns’ (defined as no change or a negative change in indicators pre and post campaign) were reported.

## Discussion

The survey demonstrated an upward trend in the number of national hand hygiene campaigns performed in EU/EEA Member States. Nineteen such campaigns had been performed between January 2000 and March 2009 and 18 more between April 2009 and December 2011 (Figure 1). Almost the same number of campaigns took place in the two and a half years post April 2009 than in the preceding nine and a half years. The reasons behind the rise in number of campaigns, particularly from 2008 onwards, were not explored. Possible reasons may include the announcement of the ‘Global Patient Safety Challenge’ at the 55th World Health Assembly in 2005, or due to the increasing burden of healthcare-associated infections on heath systems and increasing public awareness. Nevertheless, our results show that just over 50% have been evaluated, clearly indicating that hand hygiene campaigns are currently under-evaluated.

When analysing as to why 50% of campaigns were not evaluated, 20% of respondents indicated that it was too early to begin an evaluation. Indeed, although some evaluations can be initiated during the implementation phase, others such as impact evaluations are usually initiated upon nearing or completion of the campaign. This is particularly true for evaluations assessing the durability of increases in compliance rates over time, which are undertaken some time after termination of the campaign. This is particularly true for evaluations assessing the maintenance of compliance rates. These evaluations are important in the field of hand hygiene as data has shown the difficulty in maintaining achieved rates of compliance over time. This is illustrated in Figure 1, where data from 2011 illustrates evaluation rates far lower than any preceding year. It may be difficult to attain meaningful data from an evaluation of a recently implemented campaign as changes may not yet have occurred or may diminish over time as was noted earlier.

Because many of the campaigns were launched in the past few years, and given the pattern of evaluations, one could optimistically consider that the proportion of evaluated campaigns could rise by roughly 10% over the following 12–24 months. Nonetheless, the majority of respondents indicated that resource constraints, both human and financial, were the main barriers in non-evaluated campaigns (Figure 2). If resource constraints are limiting the feasibility of evaluating, then the situation may worsen in the near future as fiscal austerity in Europe places additional pressures on European public health systems.

Much data already exist on the effectiveness of hand hygiene campaigns [15,16] and enough biological and empirical evidence exists to strongly suggest that successfully implemented hand hygiene campaigns result in decreased transmission rates of HAIs [10,15,17,18]. Nonetheless, some national campaign evaluations sought to quantify this effect by assessing the effect of a campaign on HAI incidence. The England and Wales campaign for example, evaluated the effects of an increase in hand hygiene compliance rates and the quantity of alcohol-based hand rub and soap on the incidence rates of HAIs (which fell from 1.88 to 0.91 cases per 10,000 bed days for MRSA bacteraemia and 16.75 to 9.49 for cases of C. difficile infection) [19]. The quantification of impact, though extremely important and useful, may be too expensive and time consuming (and thus not appropriate) for all campaigning countries. To circumvent such issues a less comprehensive, but cheaper evaluation could be achieved by utilising targeted sampling methodologies or applying the WHO self -assessment tool at randomly selected intervention sites. Furthermore, as is practiced in other public health fields [20], an evaluation focusing on the quality of implementation as well as other tangible outcomes would be able to provide insight into the likely impact of a programme. Such methodologies, particularly if paired with incidence data for notifiable diseases such as MRSA that have the potential to be transmitted via hands, constitute a solid evaluation at a minimal cost.

As was noted, the WHO hand hygiene campaign evaluation tool allows for rapid deployment. The tool facilitates cross-country data comparisons by having standardised research methodologies and indicators. Over half of the evaluations reported utilising this tool. Developing such evaluation methodologies which use predefined criteria can reduce both human and capital resource inputs required to conduct an evaluation. The development of such tools is an important step and may be one of the most effective ways to encourage evaluations of public health programmes.

Hand hygiene campaigns were selected as the exemplary public health intervention to illustrate the frequency of evaluation for several reasons. First, hand hygiene campaigns are a behaviour change-based intervention. The strong psychosocial components mean that each intervention is subject to unique pressures and thus outcomes are not consistent. Although a strong framework is available for hand hygiene campaign evaluation, other community-based and behaviour change interventions often lack appropriate and comprehensive evaluation frameworks. Whether the results of this study, which identify a sub-optimum rate of evaluated programmes, can be extrapolated to other public health programmes remains unclear. What has been illustrated however is an increase in hand hygiene campaigns associated with a global push for safer patient care, and a strong uptake of tools for evaluation. The development of evaluation methodologies should be a focus for improving rates of evaluation.

### Limitations

The paper aimed to assess the proportion of European hand hygiene campaigns which were evaluated, characteristics of those evaluations and reasons behind non-evaluation. This was achieved, however whether the results can be extrapolated to broader and diverse European public health campaigns and programmes is unclear.

Furthermore, no clear definition of what constituted a national hand hygiene campaign or an evaluation was developed. Member states reported on their activities, thus leaving the definition of a campaign and an evaluation subject to the discretion of each country. This may have biased the results in terms of overestimating the number of campaigns and evaluations.

## Conclusion

Evaluations are an essential component of public health programmes. Their importance is widely accepted, yet application remains patchy. This investigation of evaluations amongst European national hand hygiene campaigns demonstrated this by highlighting that only half of the reported national European hand hygiene campaigns were evaluated. The availability of open-access, well-designed evaluation indicators and tools will most likely foster the employment of evaluations in the field of public health. Proactive steps should be taken at an institutional and national level to address the importance of evaluations and encourage their application. A major step would be to promote the development of standardized guidelines and other evidence-based methodologies to facilitate and support evaluations.

## Competing interests

The authors declare that they have no competing interests.

## Authors’ contributions

JRL, APM, DLM, AJ developed, supervised and managed the study. SA, OA, AB, MB, MC, CGSHHC, ACC, RC , MD, UD, SE, OG, DH, EH, JH, NK, NKS, OL, HCM, SM, MLM, CR, MS, ES, RV, RVD, NV, AV, SW provided both raw and analysed data, and edited and reviewed the manuscript in conjunction with JRL, APM, DLM, AJ. All authors reviewed and approved the final manuscript.

## Pre-publication history

The pre-publication history for this paper can be accessed here:

http://www.biomedcentral.com/1471-2458/14/131/prepub

## Supplementary Material

Additional file 1 List of relevant questions for printing Spain for the 2012 ECDC Hand Hygiene Questionnaire. Click here for file
