# Supplementary material for: The role and utilisation of public health evaluations in Europe: a case study of national hand hygiene campaigns
Source: BMC Public Health. 2014 Feb 7;14:131. doi: 10.1186/1471-2458-14-131 (PMC3931350; doi:10.1186/1471-2458-14-131)
Supplement: Additional file 1 — List of relevant questions for printing Spain for the 2012 ECDC Hand Hygiene Questionnaire. [file 1471-2458-14-131-S1.docx]

List of relevant questions for printing Spain for the 2012 ECDC Hand Hygiene Questionnaire

| **Q #** | **Question** | **Answer** | **Skips/ Instructions** |
| --- | --- | --- | --- |
|  | **Welcome to the 2012 ECDC survey**  **First and foremost we would to thank you for your cooperation and participation**  **This questionnaire is composed of two main sections**  **The first section concerns hand hygiene campaign(s) which were identified in your country by the 2009 ECDC study** [**(http://www.eurosurveillance.org/ViewArticle.aspx?ArticleId=19190**](http://www.eurosurveillance.org/ViewArticle.aspx?ArticleId=19190)**) Please use this section to provide information regarding evaluation(s) associated with those campaign(s)  *If the 2009 study did not identify any national hand hygiene campaigns in your country, you will be lead directly to the second section***  **The second section concerns any new hand hygiene campaign(s) which have been initiated in your country since (and including 2009). Please use this section to provide details about these campaign(s) as well as any associated evaluations**  **You will automatically be lead you to the appropriate starting point given the history of campaigns in your country** |  |  |
| Q.2 | Please state you sex |  |  |
| Q.3 | Please state your full name |  |  |
| Q.4 | Please state your official job title |  |  |
| Q.5 | Please state the name of your institution |  |  |
| Q.6 | Please indicate if we may contact you for further information regarding this topic |  |  |
| Q.7 | Please select which European country you are representing |  |  |
| Q.146 | **Identifying new campaigns**  ***In the 2009 ECDC study, no hand hygiene campaigns were detected in your country between 2000 - 2009.***    ***This section aims to assess whether your country has implemented any new hand hygiene campaigns since April 2009 and if these campaigns have been/ are currently being evaluated*** |  |  |
| Q.147 | Has your country had any new hand hygiene campaigns since (and including 2009)? |  | **If ‘No’ is selected, please skip to Q.242** |
| Q.148 | Please indicate the number of hand hygiene campaigns initiated since (and including 2009)? | 1  2  >2 | **If ‘1’ is selected, please skip to Q.196**  **If ‘2’ or ‘>2’ is selected please continue to Q.149** |
| Q.149 | **1st New campaign: 2009 - 2012**  *You have been directed here because you indicated the presence of two national hand hygiene campaigns in your country.*  ***Please use this section to fill in details about the first (earlier) campaign in chronological order***  *Upon completion of this section, you will be lead to an additional section where you may enter details concerning the second campaign in chronological order* |  |  |
| Q.150 | Name of Campaign |  |  |
| Q.151 | Which organisation was in charge of running the campaign? | Ministry of Health  National Health Authority  Other (please Specify) |  |
| Q.152 | Please indicate the dates of initiation (mm/yy) and completion (mm/yy) of the campaign, or indicate if it is still ongoing |  |  |
| Q.153 | As part of the campaign, please indicate which of the following activities occurred | Leaflets for healthcare workers Yes No  Posters for Healthcare workers Yes No  Websites/ e-learning tools of healthcare workers  Yes No    Training programmes Yes No  Lectures Yes No  Gadgets and merchandise (i.e badges and pens) Yes No  National Guidelines Yes No    Other (please specify) Yes No |  |
| Q.154 | Has this campaign or any of its components been evaluated/ currently undergoing evaluation? | Yes  No  Don’t know | **If ‘No’ is selected then please skip to Q.193** |
| Q.155 | What is being/ has been evaluated? | Whole campaign  Specific component (please specify) |  |
| Q.156 | Please indicate the dates of initiation (mm/yy) and completion (mm/yy) of the evaluation, or indicate if it is still ongoing |  |  |
| Q.157 | What was/ is the aim of the evaluation | Evaluate health impact  Evaluate implementation processes  Other(please specify) |  |
| Q.158 | Which organisation was/ is responsible for conducting the evaluation? | Same organisation who ran the campaign  Other (please specify) |  |
| Q.159 | Which organisation funded/ is funding the evaluation? | Same organisation who is running the campaign  Integrated into budgetary plans  Other (please specify) |  |
| Q.160 | Were key stake holders identified prior to the evaluations development process, and if so who were they? | Yes (please specify)  No  Don’t know | **If ‘No’ or ‘Don’t know’ is selected, please skip to Q.162** |
| Q.161 | Were stakeholders involved in the evaluations development process? | Yes  No  Don’t know |  |
| Q.162 | Was an evaluation plan integrated into the campaign protocol? | Yes  No  Don’t know | **If ‘No’ or ‘Don’t know’ is selected, please skip to Q.165** |
| Q.163 | Does this evaluation plan provide instructions on indicators to be used in the evaluation? | Yes  No  Don’t know |  |
| **Q.**164 | Does this evaluation plan provide guidance on the tasks, roles and responsibilities for the evaluators? | Yes  No  Don’t know |  |
| Q.165 | Briefly describe the methodology of the evaluation process |  |  |
| Q.166 | Was/ is the WHO hand hygiene evaluation toolkit used in the campaign evaluation? | Yes  No  Don’t know |  |
| Q.167 | What were the units of analysis (i.e.  hospitals assessed or interviews conducted for evaluative purposes) and how large was the sample size? *If not applicable, please explain why* |  |  |
| Q.168 | What was the outcome of the evaluation? If *not applicable please enter 'NA'* |  |  |
| Q.169 | How have the results been/ how will the results be used? |  |  |
| Q.170 | Do interim reports exist? | Yes  No  Don’t know |  |
| Q.171 | Do final documents/ manuscripts exist? | Yes  No  Don’t know |  |
| Q,172 | ***If either interim or final project documents exist****, please attach them to an e-mail addressed to* [*jonathan.latham@ecdc.europa.eu*](mailto:jonathan.latham@ecdc.europa.eu)  *Please state in the subject heading*  *Your country:*  *Interim/ final campaign documents*  *Campaign name*  ***OR if they are available online in the public domain, please provide a link to the documents*** |  |  |
| Q.173 | Have there been additional evaluations on this campaign? | Yes  No | **If ‘No’ is selected, then please skip to Q.195**  **If ‘Yes’ is selected then please continue to Q.174** |
| Q.174 | ***You have been directed here because you indicated that there have been additional evaluations on this campaign.***  ***Please complete the following section referring to details of this second evaluation*** |  |  |
| Q.175 | What is being/ has been evaluated? | Whole campaign  Specific component (please specify) |  |
| Q.176 | Please indicate the dates of initiation (mm/yy) and completion (mm/yy) of the evaluation, or indicate if it is still ongoing |  |  |
| Q.177 | What is the aim of the evaluation? | Evaluate health impact  Evaluate implementation processes  Other(please specify) |  |
| Q.178 | Which organisation was/ is responsible for conducting the evaluation? | Same organisation who ran the campaign  Other (please specify) |  |
| Q.179 | Which organisation funded/ is funding the evaluation? | Same organisation who is running the campaign  Integrated into budgetary plans  Other (please specify) |  |
| Q.180 | Were key stake holders identified prior to the evaluations development process, and if so who were they? | Yes (please specify)  No  Don’t know | **If ‘No’ or ‘Don’t know’ is selected, then please skip to Q.182** |
| Q.181 | Were stakeholders involved in the evaluations development process? | Yes No  Don’t know |  |
| Q.182 | Was an evaluation plan integrated into the campaign protocol? | Yes  No  Don’t know | **If ‘No’ or ‘Don’t know’ is selected, then please skip to Q.185** |
| Q.183 | Does this evaluation plan provide instructions on indicators to be used in the evaluation? | Yes  No  Don’t know |  |
| Q.184 | Does this evaluation plan provide guidance on the tasks, roles and responsibilities for the evaluators? | Yes  No  Don’t know |  |
| Q.185 | Briefly describe the methodology of the evaluation process |  |  |
| Q.186 | Was/ is the WHO hand hygiene evaluation toolkit used in the campaign evaluation? | Yes  No  Don’t know |  |
| Q.187 | What were the units of analysis (i.e.  Hospitals assessed or interviews conducted for evaluative purposes) and how large was the sample size? *If not applicable, please explain why* |  |  |
| Q.188 | What was the outcome of the evaluation? If *not applicable please enter 'NA'* |  |  |
| Q.189 | How have the results been/ how will the results be used? |  |  |
| Q.190 | Do interim reports exist? | Yes  No  Don’t know |  |
| Q.191 | Do final documents/ manuscripts exist? | Yes  No  Don’t know |  |
| Q.192 | ***If either interim or final project documents exist****, please attach them to an e-mail addressed to* [*jonathan.latham@ecdc.europa.eu*](mailto:jonathan.latham@ecdc.europa.eu)  *Please state in the subject heading*  *Your country:*  *Interim/ final campaign documents*  *Campaign name*  ***OR if they are available online in the public domain, please provide a link to the documents*** |  | **Skip to Q.195** |
| Q.193 | Please describe why the campaign has not been evaluated |  |  |
| Q.194 | Is/ was there an evaluation plan integrated into project documents? |  |  |
| Q.195 | **2nd New campaign: 2009 - 2012**    *You have been directed here because you indicated the presence of two national hand hygiene campaigns in your country. You have already completed a section referring to the first campaign in chronological order.*    ***Please use this section to fill in details about the second (last) campaign in chronological order*** |  | **Skip to Q.197** |
| Q.196 | **New campaign: 2009 - 2012**  *You have been directed here because you indicated the presence of one national hand hygiene campaign in your country which has been initiated since (and including) 2009.*  ***Please use this section to fill in details about this campaign*** |  |  |
| Q.197 | Name of campaign |  |  |
| Q.198 | Which organisation was in charge of running the evaluation? | Ministry of Health  National health Authority  Other (please specify) |  |
| Q.199 | Please indicate the dates of initiation (mm/yy) and completion (mm/yy) of the campaign, or indicate if it is still ongoing |  |  |
| Q.200 | As part of the campaign please indicate which of the following occurred. | Leaflets for healthcare workers Yes No  Posters for Healthcare workers Yes No  Websites/ e-learning tools of healthcare workers  Yes No    Training programmes Yes No  Lectures Yes No  Gadgets and merchandise (i.e badges and pens) Yes No  National Guidelines Yes No    Other (please specify) Yes No |  |
| Q.201 | Has this campaign or any of its components been evaluated/ currently undergoing evaluation? | Yes  No  Don’t know | **If ‘No’ or ‘Don’t know’ is selected, then please skip to Q.240** |
| Q.202 | What is being/ has been evaluated? | Whole campaign  Specific component (please specify) |  |
| Q.203 | Please indicate the dates of initiation (mm/yy) and completion (mm/yy) of the evaluation, or indicate if it is still ongoing |  |  |
| Q.204 | What was/ is the aim of the evaluation? | Evaluate health impact  Evaluate implementation processes  Other(please specify) |  |
| Q.205 | Which organisation was/ is responsible for conducting the evaluation? | Same organisation who ran the campaign  Other (please specify) |  |
| Q.206 | Which organisation funded/ is funding the evaluation? | Same organisation who is running the campaign  Integrated into budgetary plans  Other (please specify) |  |
| Q.207 | Were key stake holders identified prior to the evaluations development process, and if so who were they? | Yes (please specify)  No  Don’t know | **If ‘No’ or ‘Don’t know’ is selected, then please skip to Q.209** |
| Q.208 | Were stakeholders involved in the evaluations development process? | Yes  No  Don’t know |  |
| Q.209 | Was an evaluation plan integrated into the campaign protocol? | Yes  No  Don’t know | **If ‘No’ or ‘Don’t know’ is selected, then please skip to Q.212** |
| Q.210 | Does this evaluation plan provide instructions on indicators to be used in the evaluation? | Yes  No  Don’t know |  |
| Q.211 | Does this evaluation plan provide guidance on the tasks, roles and responsibilities for the evaluators? | Yes  No  Don’t know |  |
| Q.212 | Briefly describe the methodology of the evaluation process |  |  |
| Q.213 | Was/ is the WHO hand hygiene evaluation toolkit used in the campaign evaluation? |  |  |
| Q.214 | What were the units of analysis (i.e.  hospitals assessed or interviews conducted for evaluative purposes) and how large was the sample size? *If not applicable, please explain why* |  |  |
| Q.215 | What was the outcome of the evaluation? If *not applicable please enter 'NA'* |  |  |
| Q.216 | How have the results been/ how will the results be used? |  |  |
| Q.217 | Do interim reports exist? |  |  |
| Q.218 | Do final documents/ manuscripts exist? |  |  |
| Q.219 | ***If either interim or final project documents exist****, please attach them to an e-mail addressed to* [*jonathan.latham@ecdc.europa.eu*](mailto:jonathan.latham@ecdc.europa.eu)  *Please state in the subject heading*  *Your country:*  *Interim/ final campaign documents*  *Campaign name*  ***OR if they are available online in the public domain, please provide a link to the documents*** |  |  |
| Q.220 | Have there been additional evaluations on this campaign? | Yes  No | **If ‘No’ is selected, then please skip to Q.242** |
| Q.221 | ***You have been directed here because you indicated that there have been additional evaluations on this campaign.***  ***Please complete the following section referring to details of this second evaluation*** |  |  |
| Q.222 | What is being/ has been evaluated?  Whole campaign  Specific component (please specify) |  |  |
| Q.223 | Please indicate the dates of initiation (mm/yy) and completion (mm/yy) of the evaluation, or indicate if it is still ongoing |  |  |
| Q.224 | What is the aim of the evaluation? | Evaluate health impact  Evaluate implementation processes  Other(please specify) |  |
| Q.225 | Which organisation was/ is responsible for conducting the evaluation? | Same organisation who ran the campaign  Other (please specify) |  |
| Q.226 | Which organisation funded/ is funding the evaluation? | Same organisation who is running the campaign  Integrated into budgetary plans  Other (please specify) |  |
| Q.227 | Were key stake holders identified prior to the evaluations development process, and if so who were they? | Yes (please specify)  No  Don’t know | **If ‘No’ or ‘Don’t know’ is selected, then please skip to Q.229** |
| Q.228 | Were stakeholders involved in the evaluations development process? | Yes No  Don’t know |  |
| Q.229 | Was an evaluation plan integrated into the campaign protocol? | Yes  No  Don’t know | **If ‘No’ or ‘Don’t know’ is selected, then please skip to Q.232** |
| Q.230 | Does this evaluation plan provide instructions on indicators to be used in the evaluation? | Yes  No  Don’t know |  |
| Q.231 | Does this evaluation plan provide guidance on the tasks, roles and responsibilities for the evaluators? | Yes  No  Don’t know |  |
| Q.232 | Briefly describe the methodology of the evaluation process |  |  |
| Q.233 | Was/ is the WHO hand hygiene evaluation toolkit used in the campaign evaluation? | Yes  No  Don’t know |  |
| Q.234 | What were the units of analysis (i.e.  hospitals assessed or interviews conducted for evaluative purposes) and how large was the sample size? *If not applicable, please explain why* |  |  |
| Q.235 | What was the outcome of the evaluation? If *not applicable please enter 'NA'* |  |  |
| Q.236 | How have the results been/ how will the results be used? |  |  |
| Q.237 | Do interim reports exist? | Yes  No  Don’t know |  |
| Q.238 | Do final documents/ manuscripts exist? | Yes  No  Don’t know |  |
| Q.239 | ***If either interim or final project documents exist****, please attach them to an e-mail addressed to* [*jonathan.latham@ecdc.europa.eu*](mailto:jonathan.latham@ecdc.europa.eu)  *Please state in the subject heading*  *Your country:*  *Interim/ final campaign documents*  *Campaign name*  ***OR if they are available online in the public domain, please provide a link to the documents*** |  | **Skip to Q.242** |
| Q.240 | Please describe why the campaign has not been evaluated |  |  |
| Q.241 | Is/ was there an evaluation plan integrated into project documents? |  |  |
| Q.242 | ***You have reached the end of the survey.  If you press NEXT the survey will close, your responses and comments saved, and you will not be able to access the survey again.  Please ensure you are happy with your responses before pressing NEXT*** |  |  |

*Please do not hesitate to contact us concerning any queries or suggestions concerning this topic*

**Jonathan Ross Latham. BSc, MSc**

**Junior Consultant, Scientific Advice Coordination Section**

European Centre for Disease Prevention and Control

Tomtebodavägen 11A
171 83 Stockholm, Sweden

Tel: +46 8 5860 1013

Mob: +39 3462364036
[jonathan.latham@ecdc.europa.eu](mailto:jonathan.latham@ecdc.europa.eu)
[http://ecdc.europa.eu/](http://ecdc.europa.eu/)
